# Large and forgotten in rural Australia: assessment, attitudes and possible approaches to losing weight in young adult males

**DOI:** 10.1186/1471-2458-14-243

**Published:** 2014-03-11

**Authors:** Kumara Mendis, Tanya Forster, Karen Paxton, Karen Hyland, Jason Yelverton, Rick McLean, Joseph Canalese, Anthony Brown, Katharine Steinbeck

**Affiliations:** 1 School of Rural Health, University of Sydney, Dubbo, Australia; 2 Dubbo City Council, Dubbo, Australia; 3 Sydney Medical School, University of Sydney, Sydney, Australia; 4 Discipline of Paediatrics & Child Health, Children’s Hospital, Westmead, Australia; 5 Bathurst Rural Clinical School, School of Medicine, University of Western Sydney, Bathurst, Australia

**Keywords:** Male, Overweight, Obesity, Weight loss, Rural health, Quantitative and qualitative research, Incentives, Text messaging, SMS

## Abstract

**Background:**

Young Adult Males (YAMs) in rural Australia are poorly studied with respect to overweight and obesity. Firstly, we explored the feasibility of recruiting 17–25 year old YAMs to obtain baseline data on overweight and obesity rates, socio-demographics, nutrition, exercise and mobile phone usage. Secondly, we explored the views of YAMs with a waist measurement over 94 cm about using mobile phone text messages to promote weight loss and incentives to promote healthy lifestyles.

**Methods:**

A two-staged, mixed-methods approach was used to study obesity and overweight issues in Dubbo, a regional city in New South Wales, Australia. In Phase I, socio-demographic, health behaviour and mobile phone usage data were collected using a questionnaire and anthropometric data collected by direct measurement. In Phase II, YAMs’ views were explored by focus group discussion using a semi-structured questionnaire.

**Results:**

Phase I (145 participants): mean Body Mass Index (BMI) 25.06 ± 5.01; mean waist circumference 87.4 ± 15.4 cm. In total, 39.3% were obese (12.4%) or overweight (26.9%) and 24.1% had an increased risk of metabolic complications associated with obesity. 135 (93.1%) owned a mobile phone and sent on average 17 ± 25 text messages per day and received 18 ± 24.

Phase II (30 participants): YAMs acknowledged that overweight and obesity was a growing societal concern with many health related implications, but didn’t feel this was something that affected them personally at this stage of their lives. Motivation was therefore an issue. YAMs admitted that they would only be concerned about losing weight if something drastic occurred in their lives. Text messages would encourage and motivate them to adopt a healthy lifestyle if they were individually tailored. Gym memberships, not cash payments, seem to be the most favoured incentive.

**Conclusion:**

There is a clear need for an effective health promotion strategy for the almost 40% overweight or obese Dubbo YAMs. The high rate of text message usage makes it feasible to recruit YAMs for a prospective study in which personalized text messages are used to promote healthy behaviours. It may be important to target motivation specifically in any weight-related intervention in this group with incentives such as gym membership vouchers.

## Background

In Australia, 67.7% of males are overweight or obese [1], with a current trend of weight gain occurring at an earlier age [2]. Given this trend, there is value in an early preventative strategy addressing the overweight/obesity problem in the male 17–25 year age group [3]. Young adult males (YAMs) present a significant challenge for preventive health interventions as they consult General Practitioners less often [4], use Medicare services at less than half the rate of young women [5], and obese YAMs underestimate the risk of chronic disease [6]. Moreover, males who are obese at 20 years of age are twice as likely to pass away at any given age as their non-obese counterparts, and by the time they reach 55 years they will, on average, die 8 years earlier [7]. Swift intervention is required, not only for their own health and that of their children as they become parents, but also because they will become over-consumers of healthcare for chronic diseases within a generation [8].

The former Greater Western Area Health Service (GWAHS) region had the highest rate of overweight and obesity (Males 75.3%, Females 54.3%) in the state of New South Wales (NSW), Australia [9,10]. In the group aged 16–24 years, YAMs were significantly more overweight or obese (30.7%) than females (21.4%) [9,10]. The above data were based on self-reported height and weight using telephone surveys which may have underestimated the prevalence of overweight and obesity by as much as 23% for men and 15% for women [11].

Various programs have been devised to address overweight issues in children [3], adults [12] and females [13]. However, obesity in young males, especially in rural Australia, has been poorly researched and little is known about weight loss interventions in this age group. It has been claimed that obesity is better prevented than treated [14]. In 2000, a report from the Institute of Medicine concluded that ‘behavioural and social interventions offer great promise to reduce disease morbidity and mortality, but as yet their potential to improve has been relatively poorly tapped’ [15]. Conventional health promotion campaigns have shown a high rate of failure to promote behaviour change [16] and are particularly ineffective at addressing weight-related health behaviour change [17]. This emphasizes the need to think in novel ways about management strategies.

Mobile phone text messages [18] and financial incentives [19,20] are two new tools that have been used to promote healthy behaviours among obese individuals. Young adults are the highest users of mobile phone technology [21] and there is increasing interest in using mobile phones to deliver health improvement interventions to traditionally hard-to-reach populations [19]. Studies have shown that mobile phone and text messaging technology can be successfully utilized in Australian hospitals [22,23] and general practice settings [24] for improving healthcare. In recent years financial incentives have been associated with successful outcomes in weight loss [20,25]. With the average cost of an obesity intervention at around $AUD 6,903 per obese person [26], financial incentives may have a favourable cost benefit [27].

The objectives of this study were to explore the:

(a) Feasibility of recruiting 17–25 year old YAMs in Dubbo, NSW to obtain baseline data on the rate of overweight and obesity, and collect socio-demographic, nutrition, exercise and mobile phone usage data;

(b) Views of 17–25 year old YAMs, with a waist measurement of 94 cm or more, about being overweight or obese, and using text messages and incentives to promote healthy lifestyles

## Methods

We undertook a two-staged, mixed-methods approach to study overweight and obesity issues in Dubbo, a regional NSW city with a population of 37843 in 2006 [28].

### Phase I

#### Inclusion criteria

Entry criteria for YAMs: English speaking; aged 17 – 25 years; home post code of 2830; and owning a mobile phone.

#### Recruitment

A targeted print and digital media publicity campaign was run to raise awareness. Recruitment took place at the two main shopping malls and specific work places with high numbers of YAMs in Dubbo during three consecutive weekends in November 2006. An incentive to participate in the study was $10 of mobile phone credit.

Those who consented to participate completed a questionnaire to provide baseline information about socio-demographic, nutritional and exercise status, and mobile phone use. The questionnaire was developed locally and piloted with five YAMs and then appropriately modified. Specific questions about nutrition and eating habits were similar to those in the NSW Health Survey [8,29]. Weight (to nearest 0.1 kg) and height, hip and waist (to nearest cm) measurements were made in socks or bare feet, and in light street clothing using standard anthropometric techniques [12]. Anthropometric measurements were taken by medical students and trained staff under the supervision of a doctor. Combining waist measurement and BMI, the risk of Type 2 diabetes and cardiovascular risk was calculated according to the NHMRC guidelines [12].

Statistical data analysis was performed using SPSS software (version 15.0 SPSS Inc, Chicago IL, USA). Ethics approval was obtained from the University of Sydney Human Research Ethics Committee (Ref. No.07-2006/9015).

### Phase II

#### Inclusion criteria

Males aged between 17 and 25 years from Dubbo, NSW, who owned a mobile phone and had a waist size of 94 cm or more [12].

#### Recruitment

In 2011, we used flyers, posters, newspaper and radio advertisements, and media releases to publicise the recruitment drive for the focus group study. Recruitment was undertaken in various community settings that had a high percentage of YAMs such as local council sporting clubs, youth centres and the technical training college TAFE.

Five focus groups were conducted in total (2 – 6 participants). Three individual interviews were conducted for YAMs unable to attend a scheduled focus group. The same interviewer conducted all focus groups and individual interviews (JY).

After a review of the research literature, a broad interview schedule was developed [see Additional file 1]. According to the schedule, semi-structured interviews were conducted with both the focus groups and individual interviews. Focus group interviews lasted approximately one hour, and were moderated by a researcher independent of the research design. Interviews continued until it was felt that data saturation was achieved. The study protocol was approved by University of Sydney Human Research Ethics Committee (Ref. No. 13337).

### Data analysis

Interviews from all focus groups were recorded and transcribed before analysis began. Transcription of one-to-one interviews took place at a later stage, and these data were then compared and incorporated into the focus group data. Thematic analysis of the data took place using a constant comparative approach in order to develop the analytic categories. A three stage process was used in analysis [30,31]. Firstly, data were analysed using an open coding technique, where transcripts were read multiple times and themes were identified. The second phase involved reviewing the themes and grouping those that clustered closely together into super ordinate themes. The final stage of analysis involved selective coding, where a final search of the data was conducted to select particular sections of the data that best illustrated each theme. The three stage process of analysis was conducted independently by two researchers, and the data were then compared to increase the reliability of the results. Interviews were conducted and recorded by JY, transcribed by a professional transcription service and analysed by authors TF and KH.

## Results

### Phase I

In this study, 145 YAMs satisfying the criteria were recruited. Census data [28] indicate that there were 2,165 YAMs aged 17 to 25 years in Dubbo and therefore this sample represents 6.7% of the Dubbo YAMs population. Table 1 shows the socio-demographic profile: almost half (49.7%) still lived with one or both parents; 54.5% had completed Year 10 and 31% Year 12; 13.1% had a diploma or a degree; and only 6% had been unemployed for more than a year. Further, 75% had visited a health facility or provider during the past year.

**Table 1:** Socio-demographic profile

**Characteristic**	***n *** **(%)**
**Domestic status**	
Lives with parents	72 (49.7)
Lives with partner	27 (18.6)
Lives alone	14 (9.6)
Other	30 (20.7)
Not answered	2 (1.4)
**Total**	145 (100.0)
**Highest level of education**	
Year 10 (School Certificate)	79 (54.5)
Year 12 (Higher School Certificate)	45 (31.0)
Diploma	9 (6.2)
Graduate diploma	3 (2.1)
University degree	7 (4.8)
Not answered	2 (1.4)
**Total**	145 (100.0)
**Employment**	
Employed	121 (83.4)
Unemployed ≤ 1 month	5 (3.4)
Unemployed 2–6 months	7 (4.9)
Unemployed 7–11 months	3 (2.1)
Unemployed more than a year	9 (6.2)
**Total**	145 (100.0)
**Last visit to health care provider**	
GP	87 (60.0)
Hospital - admitted	10 (6.9)
Hospital – not admitted	6 (4.1)
Specialist consultation	6 (4.1)
Alternative medical practitioner	4 (2.8)
No visits indicated	32 (22.1)
**Total**	145(100.0)
(75% of the visits indicated were within one year)	
**Reported chronic illnesses**	99 (68.3)
Nil	28 (19.3)
Asthma	2 (1.4)
Hypertension	2 (1.4)
Depression	4 (2.7)
Other	10 (6.9)
Not answered	
**Total**	145

Anthropometric measurements are shown in Table 2. Using established criteria [12], 39.3% were overweight or obese. For waist measurement, 24.1% were over 94 cm and hence at increased risk of metabolic complications associated with obesity. Waist and BMI measurements were then combined according to the NHMRC guidelines [12] to assess obesity and the risk for Type 2 diabetes and cardiovascular disease (Table 3). There were 16 (11.0%) at ‘increased’, 4 (2.8%) at ‘high’ and 12 (8.3%) at ‘very high risk’ of diabetes and cardiovascular disease, a total of 32 (22.1%).

**Table 2:** Anthropometric measurements in young adult males in Dubbo (n = 145)

**Characteristic**	**Mean ± 1 SD or %**
Age (years)	19.2 ± 2.5
Weight (kg)	80.30 ± 17.07
Height (cm)	178 ± 6
**Waist circumference (cm)**	87.4 ± 15.4
Normal (≤ 94)	75.9%
Risk of metabolic complications (>94)	24.1%
**Body Mass Index (BMI) (kg/m**^**2**^**)**	25.06 ± 5.01
Underweight (BMI < 18.5)	2.8%
Normal (BMI 18.5 – 24.9)	57.9%
Overweight (BMI 25 – 29.9)	26.9%
Obese (BMI ≥ 30)	12.4%
Moderate (30.0 – 34)	(6.9%)
Severe (35.0 – 35.9)	(4.1%)
Very severe (> 40.0)	(1.4%)

**Table 3:** Combining waist measurement and BMI to assess obesity and the risk of type-2 diabetes and cardiovascular disease

**Classification**	**BMI**	**Waist (cm)**			**Total**
		**< 94**	**94-102**	**102+**	
**Underweight**	**< 18.5**	4 (2.8%)	0 (0%)	0 (0%)	4 (2.8%)
**Healthy weight**	**18.5-24.9**	79 (54.5%)	3 (2.1%)	2 (1.4%)^†^	84 (57.9%)
**Overweight**	**25-29.9**	24 (16.6%)	14 (9.7%)^†^	1 (0.7%)^¶^	39 (26.9%)
**Obese**	**≥ 30**	3 (2.1%)	3 (2.1%)^¶^	12 (8.3%)^§^	18 (12.4%)
					145 (100.0%)

Categories according to NHMRC Guidelines for measuring obesity and overweight [12]. Risk of type-2 diabetes and cardiovascular risk is represented as increased^†^, high^¶^ or very high^§^.

Table 4 compares some health-related data and behaviours of the study cohort with the self-reported characteristics of a similar cohort in NSW in 2006. A higher proportion of the Dubbo YAMs had an elevated BMI (39.2% vs. 31.3%; *P* < 0.06) and a lower proportion reported adequate physical activity (45% vs. 72%; *P* < 0.0001). Smoking was more prevalent than in NSW as a whole (24.1% vs. 19.1%; *P* = 0.16) but a lower proportion in Dubbo reported excess alcohol consumption (15.9% vs. 42.7%; *P* < 0.0001).

**Table 4:** Comparison of Dubbo YAMs’ health-related data and behaviours with a similar NSW YAM cohort

	**Dubbo YAMs (n = 145)**	**Comparative NSW YAM data **[32]	P – value^*^
**Age**	17 – 25 y	15 – 24 y	
**BMI greater than 25**	39.2%	31.3%	0.06
**Physical activity**	45% did some exercise on at least 5 or more days of the week (35.8% did 30 minutes or more/day)	72.37% of the males reported ‘adequate amount of exercise’	0.0001
**Fruit/vegetable consumption**			
Two servings of fruit	37.4%	47.91%	0.02
Five servings of vegetables	6.2%	3.7%	0.0001
**Smoking status**	24.1% smokers	19.1% smokers	0.16
**Excess alcohol consumption** (4 or more drinks/day)	15.9%	42.7%	0.0001

* - 2 sample Z-test to compare sample proportions.

Reported mean mobile phone talk time per day was 64 minutes (SD ± 64) with the range from 1–300 minutes a day. Half (50.4%) had a daily talk time of less than 60 minutes and 39.3% between 60 to 180 minutes. Each YAM sent an average of 17 (SD ± 25) text messages per day and received 18 (SD ± 24). Sixty percent of YAMs sent more than 10 text messages per day and 30% sent more than 20 per day.

### Phase II

Thirty YAMs with a waist circumference of 94 cm or more participated in the study. Participants had a mean age of 20.3 yrs and 8 (27%) identified themselves as Aboriginal or Torres Strait Islanders. Ten (33%) had education up to Year 10, 14 (47%) had completed high school and 5 (17%) had a diploma or a degree.

## Views on overweight and obesity

### (Direct quotes from YAMs are in Italics)

Many YAMs identified obesity as an issue for society, acknowledged the obese population was increasing, and identified that being overweight or obese was associated with a number of health related implications.

High cholesterol in the body…heart problems

Later down the track it could cause diabetes…

YAMs did not believe that their weight was affecting them at this stage in their lives.

I don’t find any real problems…I’ve always been bigger so it doesn’t really affect me much, but I see these people who are fairly obese and you can see them puffing and huffing.

The YAMs believed that in today’s society being overweight or obese has almost become the norm and there were even benefits associated with their build.

It actually helps me, being a bit bigger, playing footy now with a bit more weight about me it makes it a lot easier to hold my own on the park.

A couple of participants did feel their build was already having a negative impact on their life. These YAMs discussed the physical and emotional challenges they faced as a result of overweight and obesity.

…I have struggled for a fair while, it led to depression…

…I used to get bad reflux if I was over 100 kg…

A number of YAMs had tried to lose weight, and they discussed the challenges they faced in doing so.

…it did work, just watching what I eat and training a bit more, but I didn’t sustain it, it’s a lot harder to sustain. It’s easy to do, but it’s hard to do it over a long period of time.

…you work all day and the last thing you want to do at the end of the day is try and work out.

When they were younger they were involved in a number of sporting activities with their peers. As they reached late adolescence, they stopped playing sport.

…since I left school, I gave up the sports and I put more weight back on.

YAMs social activities instead centred on food and alcohol, which had a considerable impact on their weight and discussed the benefits associated with fast food, including the price and the convenience.

…it’s a social thing because you might go and hang out with your friends, and you go out for lunch, so you choose the quick and easy option, you go to Maccas or fast food…

…you don’t have to mess around, the mess is easy, just chuck it in the bin, don’t have to wash or wipe…

… I put on a fair bit of weight from just drinking…

While the YAMs identified how difficult it was for them to find the motivation to exercise and lose weight, they believed that they would be able to do this in the future if they needed to.

If I was ever to have kids and weight became an issue, if it ever hampered me to not be able to do things, like take them to the park, or I found it hard to get down and play with my kids, that’s when it would become an issue for me.

The YAMs identified a number of weight loss related advertising campaigns used by the Government in the past, such as ‘the advertisement of a guy running after his kid on a background of a measuring tape’. It was clear that they had not been successful in motivating the YAMs to make lifestyle changes at the time of the focus group discussions. However, this may have an effect in the future when they have kids themselves and are unable to keep up with their activities.

The YAMs agreed that providing incentives would help but did not agree on the most effective incentive they could be provided. The most popular option was gym membership vouchers.

I think gym membership would be good. It’s so expensive to do and it’s not like you can just fork it out, especially when you’ve got family, mortgages and that to pay out.

Cash as an incentive for weight loss may cause problems.

…if you’re going to give money out well they’re just going to get their money in their account and say “Hang on, I’ve got more money. Why don’t I go out and buy Maccas?”

A common theme throughout the YAMs’ discussions was the difficulties associated with working alone to try and lose weight, when those around you may not be motivated to make the same lifestyle changes.

If I say, for example, to me mates come around we’ll have a barbie and have a beer and that they all come around you know, there’d be twenty odd blokes there. If I say come to the gym with me I’d be the only bastard there.

Most YAMs preferred group training programs, so that they had the support of others in a similar position as them that would help motivate them through the challenges they faced.

Personally I need other people around me to do it…because if I’m by myself well then it’s easy to say no.

Commenting on the use of text messaging in a weight loss program aimed at motivating to lose weight, most agreed that this option could be a successful component if the messages were used well and tailored to the individual.

Like a reminder. Like a kick in the butt when you’re not feeling like following your routine. I think that’s a good idea.

…it would have to be for each individual person…people are different, do different things throughout their day…

Suggestions were made for what should be included in the message content.

Even something with your target, your current weight; your average weight loss and statistics like that just to help you or encourage you to lose that bit more or keep going with how you’re going…

…something that would push me to think, why am I eating this, why am I doing that, why shouldn’t I be doing this instead?

…did you know that if you jog on the spot 20 times you might lose X amount of kilojoules…

… an apple will wake you up three times better than a cup of coffee will…

Just think, another five weeks, five kilos, $5…

YAMs thought it would be useful to hear information about local programs and opportunities that they can become involved in.

Like Dubbo whatever the group might be called is having a training session today, come down and join the team…

…advising people on local running events… when he has his boot camps on… if there’s a special on gym membership…

YAMs also commented on the text message frequency.

Once or twice a week.

When asked if they would communicate their weight loss stories to others, particularly using social networking sites, most YAMs agreed that they would. Some said they already comment about physical activities they participate in, such as attending the gym or playing sports, on their social networking sites.

I think if it was working I’d definitely go and tell my mates…I know I’ve got a couple of mates who are embarrassed about it…if it started working for me I’d say to them straight away look this is really working, why don’t you give it a go…

…you get a lot of people check in at gyms, and “Just got back from the gym”, that sort of statement of their day.

YAMs were also asked what would motivate them to be involved in a weight loss intervention study. Responses were mixed, with some YAMs believing that incentives would be a good way to motivate people to participate, and others feeling that the opportunity to lose weight was incentive enough.

Probably a cash incentive will get most people, I think. You offer to do surveys, people usually dismiss them, they’re like door knockers, but once you offer money, people start to listen.

I think the objective of it should be almost incentive enough…

The gym membership type stuff and rewards.

## Discussion

### Phase I

This study is important because it contains direct anthropometric measures and qualitative lifestyle information in a group that has received scant attention in obesity research. In 2007, only self-reported telephone interview height and weight survey data were available for NSW YAMs [9]. Self-reported data are known to underestimate the true prevalence of overweight and obesity [10]. A validity study by Flood and colleagues of self-reported height and weight data collected by telephone in the 1997 NSW Health Survey, reported that the prevalence of overweight and obesity was underestimated by 23% for men and 15% for women [11]. When we commenced phase I in 2007, the last measured data available were from the 1995 National Health Survey [33]. The 2008 National Health Survey [1] which included direct measurement, reported in 2010, an obesity or overweight rate of 40.1% for YAMs aged 18–24 years in Australia. Our figure of 39.2% for the 17–25 year-old YAMs in Dubbo, rural NSW may be an underestimate, considering GWAHS, which encompasses Dubbo, had the highest rates of overweight and obesity of all health districts in NSW [9,10]. A limitation of our analysis is that we used a convenience sample of YAMs who attended local shopping malls and were receptive to the mobile phone credit incentives. This may have not represented the broader demographic of YAMs in Dubbo. Measurement groups anecdotally reported that some visibly larger YAMs were deterred from participation due to the measurement component of the study and thus our sample may underestimate the true prevalence of overweight and obesity in Dubbo YAMs.

In 2007, the Australian Bureau of Statistics reported that onset of weight gain appeared to be occurring at an earlier age [34]. Even though the prevalence of overweight and obesity in adults in NSW was lowest in the 16–24 year age group, males were more overweight or obese than females (30.7% vs. 21.4%) [9]. Our study population showed an even higher rate of overweight or obesity (39.3%) confirming that obesity is more prominent in rural Australia [35]. Since our sample was not representative we could not compare with the 2007 NSW Health Survey, which was based on telephone interviews.

The YAMs sent on average 17 text messages compared to a 2004 study in which 85% of participants sent fewer than 5 text messages per day [36]. Since YAMs have high usage of mobile phones and text messaging [21], the mobile phone would be a good vehicle to provide information and reinforcement regarding healthy behaviour. A systematic review of ‘healthcare via cell phones’ concluded that information and education interventions delivered through wireless mobile technology resulted in improvements in both clinical outcome (e.g. Asthma symptoms, HbA1C levels, smoking quit rates) and care processes (e.g. lower failed appointments, quicker diagnosis and treatment) [19]. The review stated that it is the first technology where industry has documented a trend towards a digital divide in the reverse, with low-income internet users spending more time on the internet than high-income users [19]. This may increase the likelihood of successfully delivering health improvement interventions to traditionally hard-to-reach populations. Feasibility studies using text messaging to promote healthy behaviours and weight loss have been reported as early as 2008 [37].

### Phase II

The follow up focus group study in Dubbo in 2010 is the first of which we are aware to report on the attitudes of rural, young adult males towards being overweight or obese and attempting weight loss. A focus group study provides novel and useful information for the planning of weight loss interventions in this largely under-researched but important group.

While YAMs acknowledged that being overweight and obese was a growing societal concern with a number of health related implications, they did not feel that being overweight was affecting them personally. It is thus not surprising that motivation towards weight loss was a clear issue for them. YAMs indicated they would be concerned about losing weight only if something drastic occurred in their lives, although with such events phrased in the future tense. Individually tailored text messages were considered a potential motivator, as was the incentive of a gym membership voucher. Cogent reasons were provided as to how these two elements might promote weight loss.

Young people aged 17-24 have minimal contact with health services [4,5] and perceive the threat of chronic illness as irrelevant [8]. YAMs in our study echoed that chronic illness is something for the future and is irrelevant for them at present, an attitude not surprising for their developmental stage [38]. In contrast to the earlier findings [4,5], 78% of the YAMs in our cohort had met with a healthcare provider recently, with 75% of these visits occurring within the past year. The YAMs felt they would be motivated to lose weight once they thought their weight was becoming an issue or interfering in their lives, therefore taking a reactive approach rather than using any preventative measures. Planned interventions targeting lifestyle changes in YAMs would need to identify motivating factors other than the future threat of chronic illness. Education regarding more immediate health concerns may be of use, such as a reported increase in sexual dysfunction amongst obese males [39].

Interestingly, the YAMs stated that they did not feel that being overweight or obese was a social issue for them personally. This is in contrast with previous research on adults, with particular focus on females, conducted by Thomas and colleagues [40] which highlighted the social isolation participants felt as a result of their build. Discrepancies between the results of these two studies reinforce the importance of conducting gender and age specific research. Lewis and colleagues found that mildly obese individuals felt little need to change their health behaviours, believed they could lose weight if they needed to, distanced themselves from the word obesity, and stigmatized those larger than themselves [41]. YAMs involved in this study had similar perceptions to the participants in the study by Lewis and colleagues which mainly included males. Men prefer to think they are dieting for legitimate reasons [41] and think women who diet as doing so for cosmetic reasons, as in the research of Thomas and colleagues [40]. Thus discrepancies between the results of these two papers could be solely the result of issues relating to gender. The gender difference of weight loss interventions is noted in a systematic review in which it was observed that men participate more in exercise training programs while women undergo more diet and behavioral intervention programs [42].

Unexpectedly, YAMs requested gym membership vouchers instead of cash payments. The cost of gym membership should be much more acceptable for human ethics committees and funding agencies than cash incentives [43]. Although research has indicated the potential of mobile phone technology in promoting health based interventions [18], we have not been able to identify any published research on the use of mobile phone technology in the difficult to engage Australian YAMs. YAMs in this study agreed that such an intervention would suit them and provided useful suggestions about message content. Given the YAMs described how socially isolating they find making healthy lifestyle changes, such as beginning physical exercise routines, text messages have the potential to provide the motivation and social support that YAMs do not feel they can get from their surrounding peer or family group.

As the study aimed to use participatory methods to inform future rural research, the sample sizes were small and participants recruited from a single rural community, which may not be generalisable to other YAMs populations. The high (16.5%) indigenous population of Dubbo, as compared to the Australian national average of 2.5%, may be a factor that may work against the generalisability. Further, when we recruited overweight or obese YAMs for this phase, waist measurements of 94 cm or more were used as the inclusion criteria rather than a BMI above 25. A systematic review about weight loss interventions in 18–25 year olds highlighted recruitment as a major issue in young people, with some studies stating that they were unable to recruit the number of participants originally planned [42]. However rural obesity research, particularly that regarding YAMs, is a priority. Given the sensitive nature of the research topic, it may be that those who did lack confidence about their body image, or were facing associated physical or emotional challenges, chose not to participate in the research discussions. The different size of the focus groups, group dynamics and social desirability processes are likely to play a role in research of this nature. The payment offered may also have affected the reason some participated.

## Conclusion

Approximately 40% of YAMs in Dubbo, a rural city in NSW, Australia are overweight or obese. Nearly 40% had not visited a general practitioner recently and 22% did not visit any healthcare provider within the past year, thus missing a traditional means of delivering health promotion messages. There is a clear need for an effective form of health promotion strategy for this under-researched group. The high rate of text message usage makes it feasible to recruit YAMs for a prospective study in which messages to promote healthy behaviours can be delivered via SMS. Furthermore, important attitudinal information has been obtained which indicates that it may be important to target motivation specifically in any weight-related intervention with incentives such as gym membership, group participation and well-designed, YAM-friendly text messages.

## Abbreviations

BMI: Body mass index; GWAHS: Greater Western Area Health Service; NHMRC: National Health and Medical Research Council; NSW: New South Wales; SMS: Short message service; YAM: Young adult male.

## Competing interests

The authors declare that they have no competing interests.

## Authors’ contributions

KM is the study leader and made a substantial contribution to the conception, design and implementation of the study and writing the first draft of Phase I, II and combined study. RM and JC contributed to Phase I design and implementation. AB contributed to the Phase I study design and focus groups. TF provided research support during data collection, coded and analysed qualitative data and drafted the manuscript for the focus group section. KH assisted with the qualitative data analysis. KP assisted with ethics approval, study design and implementation for Phase II. JY and KM conducted focus groups and interviews. RM, AB, JC contributed to writing up the manuscripts. KS contributed to the conception and design of phase II and rewriting of the combined manuscript. All authors read and approved the final manuscript.

## Pre-publication history

The pre-publication history for this paper can be accessed here:

http://www.biomedcentral.com/1471-2458/14/243/prepub

## Supplementary Material

Additional file 1 **Focus Group Schedule.** Lists the schedule of questions posed to participants in the focus groups and interviews. Click here for file
