# Supplementary material for: Large and forgotten in rural Australia: assessment, attitudes and possible approaches to losing weight in young adult males
Source: BMC Public Health. 2014 Mar 11;14:243. doi: 10.1186/1471-2458-14-243 (PMC4008138; doi:10.1186/1471-2458-14-243)
Supplement: Additional file 1 — Focus Group Schedule. Lists the schedule of questions posed to participants in the focus groups and interviews. [file 1471-2458-14-243-S1.pdf]

THE UNIVERSITY OF SYDNEY

School of Rural Health
Sydney Medical School

ABN 15 211 513 464

INVESTIGATORS
Dr Kumara Mendis
Associate Professor Anthony Brown
Professor Katharine Steinbeck
Mrs Karen Paxton

11 Moran Drive
PO Box 1043
DUBBO NSW 2830
Telephone: +61 2 6885 7977
Facsimile: +61 2 6885 7979

Email: kumara.mendis@sydney.edu.au

TITLE: **Dubbo Young Adult Males Focus Group and Interview Study (DYFIS): Will text messages and incentives help promote weight loss?**

FOCUS GROUP and INTERVIEW QUESTIONS

Interview protocol (questions, discussion – prompts (PRT) & probes (PRO)

1) **What are your views about overweight/obesity (OWOB)?**
   PRT/PRO
   a. Is OWOB a problem? (health/ social problem?)
   b. How relevant is OWOB to you?
   c. Have you ever tried to lose weight? What did you do?/ how well did it work?
   d. What do you think causes OWOB?
   e. Would OWOB cause any health problems for young men like you?
2) **How do you think we could promote**
   a. Healthy eating, regular exercise, other measures
3) **What will motivate you to lose weight?**
   PRT/PRO
   a. What would influence you to lose weight?
   b. What do you think is a realistic weight loss per month if one is actively trying to lose weight?
   c. What type of incentives would you be interested? - e.g. movie tickets, gift vouchers, running shoes, gym memberships, cash
   d. If you are interested in cash payment as incentives what (reasonable) amount would be adequate
4) **What do you think about using text messages to promote weight loss?**
   PRT/PRO
   a. What kind of TEXT messages would you be interested?
   b. Text messages – a good way of communicating about healthy eating?
   c. Text messages – a good way of communicating about regular exercise?
   d. How frequent would you like to receive health promotion Text message?
   e. Would you let others know if you were doing something positive regarding your health? e.g. exercising, having fruits (using TWITTER?)
   f. Would it be a problem if we call you on your mobile regarding health promotion
5) **How can we recruit people like you to a study similar to this?**
   PRT/PRO
   a. What would make you want to be involved in a study like this?
   b. Would you tell a friend about this study?
   c. Where can we recruit young adult males for a study like this?

6) **Would you like to know the results of this Focus Group Study? How?**
7) **Any other issues you would like to raise/discuss?**
